# Editorial: Drought stress in legumes

**DOI:** 10.3389/fpls.2022.1026157

**Published:** 2022-09-16

**Authors:** Ana Laura Furlan, Esther M. Gonzalez, Swarup Roy Choudhury, Santiago Signorelli

**Affiliations:** ^1^ Institute of Agrobiotechnological Research, (INIAB-CONICET), Faculty of Exact, Physical-Chemical and Natural Sciences, National University of Río Cuarto, Río Cuarto, Córdoba, Argentina; ^2^ Institute for Multidisciplinary Research in Applied Biology (IMAB), Public University of Navarra, Pamplona, Spain; ^3^ Department of Biology, Indian Institute of Science Education and Research (IISER) Tirupati, Tirupati, Andhra Pradesh, India; ^4^ Food and Plant Biology group, Department of Plant Biology, School of Agriculture, Universidad de la República, Montevideo, Uruguay; ^5^ School of Molecular Sciences, The University of Western Australia, Crawley, Australia

**Keywords:** water stress, tolerance, soybean, alfalfa, transcriptome, transcription factors

Plants are challenged by diverse environmental constraints, among which drought stress is increasingly important. Meteorological models predict an increase in the areas prone to drought in the future. Legumes are important sources of fiber, oils, and protein, constituting an essential amenity in the global economy. Additionally, legumes contribute to nitrogen input in the biosphere due to their ability to establish symbiotic interactions with diazotrophs, collectively named rhizobia. Therefore, efforts to decipher the molecular, metabolic, physiological, and agronomic responses are crucial contributing novel strategies to aid drought tolerance in legumes.

This Research Topic contains articles either providing new findings or discussing the latest research concerning drought research in legumes, including one mini-review on soybean tolerance to drought (Arya et al.) and seven original research papers dealing with strategies to confer drought tolerance such as priming (Zhou et al.); studies on intraspecific variation in traits associated with drought tolerance (Prince et al.); the analysis of water use efficiency under terminal drought (Polania et al.); and the contribution of a legume dehydrin to drought tolerance (Sun et al.); the functional characterization of a LOX gene family (Mou et al.); the functional analysis of a soybean APETALA2/ETHYLENE RESPONSIVE FACTOR (AP2/ERF) (Wang et al.); and a study on AP2/ERF gene family in a tolerant desert legume (Zhao et al.).

Briefly, Arya et al. outline the main strategies used to develop drought-tolerant soybeans, or as they proposed, drought-smart soybeans. Marked assisted breeding, genetic engineering, genome editing, and microbial inoculants are some strategies they proposed to breed drought-tolerant soybeans. Moreover, they summarized QTLs identified for particular traits of interest. Finally, they provided a list of references in which the expressions of specific genes have been related to traits often associated with enhanced drought tolerance.

Zhou et al. showed that γ-aminobutyric acid (GABA) priming of white clover seeds significantly alleviates the germination inhibition provoked by water stress. The exogenous application of GABA reinforced the antioxidant machinery, favored the accumulation of the dehydrin proteins, and enhanced the expression of DREB (dehydration-responsive element binding)-type transcription factors in seeds. The authors proposed GABA as a signaling molecule regulating physiological and biochemical responses to water stress.

Prince et al. evaluate morphological and physiological characteristics of roots and shoots under progressive drought (mesocosms) and rainfed conditions (open-field) in alfalfa. The authors identified several traits that influence biomass production under water limitations. Some genotypes had beneficial alleles to maintain stomatal conductance and chlorophyll content and improve productivity. Thus, identifying these alleles is expected to enable the development of drought-adapted alfalfa cultivars.

Polania et al. phenotyped water deficit responses and water use in tepary beans, a drought-resistant bean, and two common beans, one resistant and the other susceptible to terminal drought. The authors found two different water use strategies in the drought-resistant genotypes. In common bean, early stomata closure, inhibition of stomatal development, and growth limitation were the strategies found to ensure water conservation, whereas, in tepary bean, prolonged stomatal opening and higher carbon fixation, combined with unaltered stomata distribution, lead to higher biomass accumulation. This knowledge can be meaningful for breeding programs.

*Ammopitanthus nanus* is an ancestral legume species that grows in arid desert habitats in Central Asia. Sun et al. investigated this plant to elucidate abiotic stress tolerance mechanisms. The authors identified a drought-inducible gene in an RNA-seq library, a dehydrin ortholog in *Arabidopsis* (AnDHN), and transformed *Arabidopsis* plants to overexpress this gene. The overexpressing lines had an increased germination capacity, higher relative water content, longer root length, and reduced oxidative damage when subjected to cold, osmotic, and saline stress, suggesting improved abiotic stress resistance.

Mou et al. performed a genome-wide identification of the lipoxygenase (LOX) gene family in cultivated peanuts (*Arachis hypogaea*) and its wild-type progenitors (*Arachis duranensis* and *Arachis ipaensis*). They identified 72 putative peanut *LOX* genes localized in different chromosomes, and genome duplication analysis suggested how segmental duplication events expanded *LOX* genes in peanut. Cis-acting element analysis indicated that LOX genes might be involved in transcription, cell cycle, development, hormonal, and stress responses. Further investigation confirmed that *AhLOX29* has a role in drought response as overexpression of *AhLOX29* in *Arabidopsis thaliana* exhibited increased chlorophyll, proline, and superoxide dismutase activity.

Wang et al. identified AP2/ERF (APETALA2/ETHYLENE RESPONSIVE FACTOR) genes encoding transcription factors (TF) in the soybean genome and characterized the target gene *GmAP2/ERF144*, which was identified to be markedly up-regulated by drought and salt stress through transcriptomic analysis. Overexpression of *GmAP2/ERF144* increased drought resistance, and networking analysis revealed its coordination with other TF-encoding gene families. Therefore, this study contributes to understanding drought resistance mechanisms in soybean and, ultimately, cultivating drought-resistant varieties.

Zhao et al. performed a genome-wide investigation of the AP2/ERF transcription factor family in the desert legume *Eremosparton songoricum* to understand their evolution and role in drought stress response. Gene structure analysis showed that these genes were unevenly distributed in 8 chromosomes, with various duplication events. The promoter region displayed 116 cis-responsive elements related to distinct biological functions. Protein-protein interaction network analysis revealed the interaction with 160 other transcription factors. These genes were shown to significantly increase under water deficit conditions by transcriptomic and qRT-PCR data, indicating their role in drought tolerance.

The research outlined here reveals that phenotyping and transcriptomic data are valuable tools to identify relevant roles of physiological, metabolic, and molecular (mainly *via* transcription factors) traits in regulating water stress response in legumes, ultimately determining their tolerance.

## Author contributions

AF, SS, EG, and SC contributed to the writing of this editorial. All authors revised and improved the final version of the editorial.

## Funding

AF work was financed by PICT-2020-02926 from FONCYT. Research in SRC lab is supported by DBT-Ramalingaswami Re-entry Fellowship (BT/RLF/Re-entry/01/2018) and STARS Research Grant (MoE/STARS-1/508). SS work is supported by the CPR scheme of the International Centre of Genetic Engineering and Biotechnology (ICGEB) ICGEB_URY21_04_EC_2021 and the CSIC I+D program of CSIC (Uruguay) CSIC_I+D_2020_21.

## Acknowledgments

The Guest Editors would like to thank all the authors who contributed to this Research Topic. AF is thankful to National Scientific and Technical Research Council (CONICET, Argentina), SS is thankful to the Uruguayan National System of Researchers (SNI, Uruguay) and the Basic Research Development Program (PEDECIBA, Uruguay). EG is thankful to the Navarra Government and the Public University of Navarra for research funding (Spain). SC is thankful to the Indian Institute of Science Education and Research (IISER), Tirupati.

## Conflict of interest

The authors declare that the research was conducted in the absence of any commercial or financial relationships that could be construed as a potential conflict of interest.

## Publisher’s note

All claims expressed in this article are solely those of the authors and do not necessarily represent those of their affiliated organizations, or those of the publisher, the editors and the reviewers. Any product that may be evaluated in this article, or claim that may be made by its manufacturer, is not guaranteed or endorsed by the publisher.

